# New generation indirect calorimeters for measuring energy expenditure in the critically ill: a rampant or reticent revolution?

**DOI:** 10.1186/s13054-016-1315-4

**Published:** 2016-06-05

**Authors:** Elisabeth De Waele, Patrick M. Honore, Herbert D. Spapen

**Affiliations:** ICU Department, Universitair Ziekenhuis Brussel, Vrije Universiteit Brussel, 101, Laarbeeklaan, 1090 Brussels, Belgium

**Keywords:** Nutrition, Indirect calorimetry, Intensive care

## Abstract

To lower the risk of incorrectly feeding critically ill patients, indirect calorimetry (IC) is proposed as the most ideal method to evaluate energy expenditure and to establish caloric goals. New IC devices are progressively introduced but validation of this new generation remains challenging and arduous.

Nutrition has definitely forged a place amidst the therapeutic armamentarium of the intensive care unit (ICU) [1, 2]. “Nutrition pharmacology” has developed into an intrinsic ICU subspecialty and knowledge on “critical care nutrition” is growing steadily [3]. Experts agree that energy-protein targeting is of cardinal importance in fragile and often malnourished ICU patients [4]. Adequate provision of calories over time is also linked to an improved clinical outcome [5].

Today, it is evident that a correct estimation of resting energy expenditure (REE) is indispensable within an ICU nutritional care plan. Equations for calculating REE often generate insufficiently precise or poorly reproducible results in critically ill patients [6, 7]. Indirect calorimetry (IC) may more accurately predict energy requirements and is actually recommended for use in this population [8]. For decades, the Deltatrac counted as the “gold standard” metabolic monitor for measuring REE in a critical care setting. The Deltatrac gained this status because it harvested measurements of oxygen consumption (VO_2_) and carbon dioxide production (VCO_2_) in mechanically ventilated patients that were equivalent to those obtained by mass spectrometry [9]. Unfortunately, production of the Deltatrac device has ceased completely. As a result, we are now facing a surge of “new generation” ICs aiming to fill in this gap. These devices rely on breath-by-breath technology for measuring gas exchange, which differs from the mixing chamber method used by the Deltatrac. Initial experience comparing novel ICs with the Deltatrac in spontaneously breathing subjects showed good precision and acceptable bias [10, 11]. However, mechanically ventilated ICU patients represent a particular challenge. Patient–ventilator interactions, either involuntarily but also increasingly indulged in modern ventilation strategies, may significantly affect or perturb gas exchange patterns and result in inconsistent measurements. In addition, novel ICs have not been extensively tested in thermogenically “unstable” conditions created by catecholamine treatment, varying sedation levels, more frequent use of continuous extracorporeal, including renal, supportive therapy, and differences in type and quantity of feeding. Studies on validation of novel IC instruments in mechanically ventilated patients have been disappointing. A study comparing the Deltatrac with the Medgraphics Ultima calorimeter showed acceptable bias but poor precision for measuring VO_2_ [12] and poor agreement was found between the Deltatrac and the Quark RMR, M-COVX, and Evita 4 monitors [13, 14].

In this issue of *Critical Care*, Sundström Rehal et al. present an elaborate study that underscores the complexity and pitfalls of metabolic measurement in the ICU [1]. Within a robust methodological framework, these investigators compared two new generation ICs (E-sCOVX and Quark RMR) with the Deltatrac. Both modern ICs systematically overestimated VO_2_ and VCO_2_ and showed high variability in REE assessment. Unlike Sundström Rehal et al., we believe that the degree of overestimation and observed lack of precision seriously questions whether these instruments have a compelling role in daily metabolic measurement. Results must also be interpreted within the constraints of a rigorous study protocol which may not be easily applicable in daily ICU routine. Nonetheless, the work of Sundström Rehal et al. holds an outspoken claim to further invest in appropriate validation studies and to foster research into functional improvement of existing devices or even the development of a specific ICU calorimeter.

## Abbreviations

ICU: intensive care unit
REE: resting energy expenditure
VO_2_: oxygen consumption
VCO_2_: carbon dioxide production
